# Baseline MRI findings as predictors of hypopituitarism in patients with non-functioning pituitary adenomas

**DOI:** 10.1530/EC-21-0386

**Published:** 2021-10-11

**Authors:** Reem Al Argan, Abdulaziz Ramadhan, Ramanakumar V Agnihotram, Jeffrey Chankowsky, Juan Rivera

**Affiliations:** 1 Endocrine Section, Department of Internal Medicine, College of Medicine, Imam Abdulrahman Bin Faisal University, King Fahd Hospital of the University, Khobar, Eastern Province, Saudi Arabia; 2 Division of Endocrinology and Metabolism, Department of Medicine, McGill University Health Center, Montreal, Quebec, Canada; 3 Statistician, Research Institute-McGill University Health Center, Montreal, Quebec, Canada; 4 Department of Diagnostic Radiology, McGill University Health Center, Montreal, Quebec, Canada

**Keywords:** non-functioning pituitary adenoma, hypopituitarism, pituitary MRI, secondary adrenal insufficiency, central hypothyroidism

## Abstract

Hypopituitarism tends to occur in large pituitary adenomas. However, similar tumors could present with strikingly different hormonal deficiencies. In this study, we looked at MRI characteristics in non-functioning pituitary adenomas (NFPA), which could predict secondary adrenal insufficiency (SAI) and central hypothyroidism (CHT). We reviewed the files of patients with NFPA attending our clinic. Tumor size, invasiveness, MR-signal intensity, and gadolinium enhancement in preoperative MRI were recorded along with documented presurgical hypopituitarism profile. Logistic regression was used to predict SAI, CHT, or both (SAI/CHT) based on MRI and demographic parameters. Receiver operating characteristic curves were used to determine their diagnostic utility. One hundred twenty-one patients were included in the study. Older age (*P = *0.021), male sex (*P = *0.043), stalk deviation (*P* < 0.0001), contrast enhancement (*P = *0.029), and optic chiasma compression (*P = *0.012) were associated with SAI/CHT. Adenoma vertical height, largest diameter, and estimated volume were also strongly associated with SAI/CHT (*P* < 0.0001). These associations remained significant in a multivariate analysis. No tumor smaller than 12 mm in vertical height, 17 mm in largest diameter, or 0.9 cm^3^ in volume was associated with SAI/CHT. At cut-off ≥18 mm for vertical height, ≥23 mm for largest diameter, and ≥3.2 cm^3^ the sensitivity was around 90–92% for detecting SAI/CHT. Only vertical height was significantly associated with any one or more pituitary hormonal deficit (*P = *0.001). In conclusion, adenoma size, independent of the measurement used, remains the best predictor of SAI/CHT in NFPA. Dynamic testing to rule out SAI is probably indicated in adenomas larger than 18 mm vertical height, 23 mm largest diameter and 3.2 cm^3^ adenoma volume.

## Introduction

Pituitary adenomas (PA) are frequent intracranial neoplasms (1, 2). About half of these tumors are non-functioning pituitary adenomas (NFPA), meaning that they do not secrete a hormonal product capable of causing a clinical syndrome (3, 4, 5). They may, however, cause pituitary hormonal deficits (hypopituitarism) which are associated with increased morbidity and mortality (6, 7). Therefore, making a timely diagnosis of hypopituitarism is very important.

Pituitary adenomas have been historically classified as microadenomas (<1 cm) or macroadenomas (>1 cm) according to their largest diameters (1). One of the clinical implications of this classification is that the risk of hypopituitarism is considered minimal for microadenomas (7, 8, 9). The Endocrine Society guidelines for the management of pituitary incidentalomas (7) recommends 'routine testing for hypopituitarism in macroadenomas and larger microadenomas', for example, 6–9 mm, but not necessarily in smaller microadenomas, as larger lesions 'seem more likely to be associated with hypopituitarism'.

Assessing the risk of hypopituitarism is of particular importance with regards to adrenocorticotropic hormone (ACTH) and thyroid stimulating hormone (TSH) deficiency, which results in secondary adrenal insufficiency (SAI) and central hypothyroidism (CHT). Untreated SAI and CHT may lead to life-threatening complications, particularly during stress. Additionally, their diagnosis often requires the use of dynamic endocrine tests, which may require specially trained personnel (10, 11).

MRI is the imaging of choice for the evaluation of pituitary adenomas (12, 13). In a series by Zada *et al*., MRI features were reported to be predictors of adenoma subtype (12). In another study, the degree of enhancement on T1-weighted sequences correlated with the proportion of hormone-positive cells (13). Some MRI features have been reported as predictors of response to treatment in functional adenomas (14, 15). In regards to tumor size, various tumor diameter cut-off has been proposed as predictors of specific pituitary hormone deficiencies (16).

The current report presents our findings in a group of unselected patients with NFPA. We aim to establish which tumor characteristics on gadolinium-enhanced sellar MRI best predict SAI and CHT. In addition to determining optimal cut-off tumor size to recommend dynamic testing for SAI, we explore other MRI features which may enhance the prediction of SAI and CHT.

## Methods

Adult patients with NFPA attending the endocrine tumor clinics of the Royal Victoria Hospital in Montreal (Quebec, Canada) were eligible to participate. Patients were added to our database in the order they were seen between 2009 and 2018.

Diagnosis of NFPA was based on the confirmation of adenoma by MRI defined as: (i) Microadenoma which is an adenoma <1.0 cm suggested by hypo- or isointense lesion relative to normal pituitary tissue on unenhanced T1-weighted images which remains hypointense after contrast administration or enhance in delayed image (17). (ii) Macroadenoma which is an adenoma of ≥1.0 cm suggested by enlarged sella turcica with varying T1/T2 signal intensity according to necrotic and/or hemorrhagic areas with possible extension superiorly toward the optic pathways, laterally to the cavernous sinuses or inferiorly to the sphenoidal sinus (18). Non-functioning status was confirmed based on the lack of clinical and biochemical evidence of pituitary hormonal overproduction (except for mild hyperprolactinemia attributable to pituitary stalk effect). However, histological diagnosis was not available in all cases since not all of them were operated on especially when there was no indication for surgery like in microadenomas.

All patients charts were reviewed for preoperative hormonal tests, MRI reports, and images. The primary outcome of interest was SAI/CHT before pituitary adenoma-related medical or surgical treatment was initiated. We also looked at the presence of any one or more pituitary hormonal deficiency at diagnosis.

### Imaging analysis

Each patient baseline gadolinium-enhanced MRI of the sella turcica before any medical or surgical therapy was reviewed. Measurements were made with standard electronic tools (calipers) available on the IntelePACS image viewing software (Intelerad Medical Systems, Canada). The largest craniocaudal (CC or vertical height), transverse (T), and anteroposterior (AP) diameters of the pituitary adenoma were measured on sagittal, coronal, and axial contrast-enhanced images. Adenoma volume was calculated from the measured dimensions using a simplified formula for ellipsoid spheres: (AP × CC × T)/2.

Additionally, the following features of each adenoma were recorded: cavernous sinus invasion, suprasellar extension, signal intensity on T1 and T2, homogeneity, presence of cystic components, stalk deviation, and gadolinium enhancement. The suprasellar extension was graded according to the proximity to the optic chiasma (OC) as follows: 0 (away from optic chiasma); 1 (in contact with the optic chiasma, but without displacing it); or 2 (causing displacement of optic chiasma). Cavernous sinus (CS) invasion was graded as 0 (absent) or 1 (present, including Knosp 1–4). Signal intensities were recorded as either hypo-, iso-, or hyperintense as compared with the brain cortex. Stalk deviation refers to the lateral deviation from the vertical on coronal views, and it was reported as present or absent; when the suprasellar extension of the tumor caused the pituitary stalk to be not visible, it was graded as deviated stalk. Gadolinium enhancement was recorded as present or absent as assessed by eyeball comparison with pre-contrast images. All of these MRI features were tested for their association with SAI/CHT and with one or more any pituitary hormonal deficit.

### Hormonal analysis

For each patient, we reviewed their baseline pituitary hormonal profile as well as the results from stimulation tests, before any medical or surgical treatment. Secondary adrenal insufficiency (SAI) was defined as basal 07:00–09:00 h cortisol (AM cortisol) below 100 nmol/L, or 1-µ cosyntropin stimulated cortisol below 400 nmol/L or peak cortisol during insulin-induced hypoglycemia test below 500 nmol/L. Central hypothyroidism (CHT) was diagnosed in the presence of low-free T4 and low or inappropriately normal TSH, in the absence of systemic illness. Secondary hypogonadism was diagnosed based on low testosterone with low or inappropriately normal lutenizing hormone (LH)/ follicle stimulating hormone (FSH) in men and hypogonadotropic amenorrhea in women, when other causes were excluded. In post-menopausal females, secondary hypogonadism was diagnosed based on inappropriately low gonadotropins for their menopausal status. Diagnosis of growth hormone (GH) deficiency was based on low insulin like growth factor-1 in the setting of three other pituitary hormonal deficiencies and/or low GH response to glucagon stimulation test (GH peak <1 µL) or insulin-induced hypoglycemia test (GH peak <5 µ/L). We recorded the following items as presents or absent: SAI, CHT, SAI, and CHT, at least one (any) pituitary hormonal deficit, and multiple pituitary hormonal deficiency.

### Statistical analysis

Descriptive analysis was conducted on demographic and clinical characteristics stratified by presence of SAI and CHT. Ordinal data were presented in the form of frequencies and percentages, while continuous data were reported in the way of means and standard deviations. Rank-sum and chi-square tests were used to determine their statistical significance at 5% level of tolerance. To examine the utility of adenoma vertical height, largest diameter, and volume as predictive factors, logistic regression was conducted. Odds ratios (OR) and 95% CIs were calculated after adjusting for prior and empirical confounders (sex, invasiveness, homogeneity, enhancement, cystic changes, and OC compression). Receiver operating characteristic (ROC) curves were used to determine the diagnostic utility of the variables investigated by optimizing the sensitivity and specificity in predicting SAI and CHT.

## Results

### Demographics and MRI findings

After approval by McGill University Health Center Research Ethics Board, a total of 165 patient charts were reviewed; and 44 patients were excluded because of incomplete preoperative hormonal assessment or if a gadolinium-enhanced MRI of the sella was not available. The remaining 121 patients were included in this analysis. Forty-eight patients (39.7%) were females. Mean age was 56 years in females and 57 years in males. Younger patients (under 55 years old) represented the larger group (43.4%); meanwhile, 26.2% were 55–64, 15.6% between 65 and 74, and 14% were 75 years old and older (Table 1A).

**Table 1:** Patients demographics and theirs adenomas MRI characteristics distribution with or without SAI and/or CHT.

Variable	A	B		*P* value (for difference in B)
	All patients	Patients without SAI and/or CHT	Patients with SAI and/or CHT	
Sex	*n* (%)			
Female	48 (39.7)	32 (47.8)	16 (29.6)	
Male	73 (60.3)	35 (52.2)	38 (70.4)	*0.043*
Mean age (s.d.)	56.9 (14.4)	53.4 (16.3)	60.4 (12.1)	0.0206
Age group	*n* (%)			
<55	52 (42.9)	36 (53.7)	16 (29.6)	
55–64	32 (26.2)	11 (16.4)	21 (38.9)	
65–74	19 (15.6)	11 (16.4)	8 (14.8)	
75+	18 (14.8)	9 (13.4)	9 (16.7)	*0.018*
Mean tumor size	Mean (s.d.)			
Vertical height (mm)	23.4 (7.99)	19.9 (9.5)	27.7 (8.8)	*<0.0001*
Largest diameter (mm)	25.3 (9.5)	22.2 (9.4)	29.2 (8.3)	*<0.0001*
Linear volume (cm^3^)	6.48 (6.19)	4.7 (4.5)	8.6 (7.3)	*<0.0001*
Stalk deviation	*n* (%)			
Absent	13 (10.7)	13 (19.4)	0	
Present	107 (88.4)	54 (80.6)	53 (100)	*< 0.001*
CS invasion	*n* (%)			
Absent, Knosp 0–1	48 (39.7)	28 (41.8)	20 (37)	
Present, Knosp 2–4	73 (60.3)	39(58.2)	34 (64.1)	0.595
Contact with OC				
None	32 (26.44)	25 (37.3)	7 (13.2)	
Minimal	27 (22.31)	15 (22.4)	12 (22.6)	
Displaced	61 (50.41)	27 (40.3)	34 (64.1)	*0.012*
MRI intensity on T1 – WI				
Hypointense	24 (19.8)	18 (26.9)	6 (11.32)	
Isointense	87 (71.9)	44 (65.7)	43 (81.1)	
Hyperintense	9 (7.4)	5 (7.5)	4 (7.5)	0.122
MRI intensity on T2 – WI				
Hypointense	7 (5.8)	5 (7.5)	2 (3.8)	
Isointense	27 (22.31)	13 (19.4)	14 (26.4)	
Hyperintense	86 (71.1)	49 (73.1)	37 (69.8)	0.452
Heterogenous signal intensity				
Absent	46 (38)	25 (37.3)	21 (38.9)	
Present	75 (62)	42 (62.7)	33 (61.1)	0.859
Enhancement post-gadolinium				
Absent	15 (12.4)	13 (19.4)	2 (3.7)	
Present	103 (85.1)	52 (77.6)	51 (94.4)	
Missing data	3 (2.4)	2 (3)	1 (1.9)	0.29

CHT, central hypothyroidism; CS, cavernous sinus; OC, optic chiasam; SAI, secondary adrenal insufficiency; WI, weighted image.

Ninety-three percent of patients (*n* = 112) had macroadenomas (largest diameter ≥1 cm). The mean adenoma vertical height was 23.8 mm (range 5–47 mm); the mean largest adenoma diameter was 25.7 mm (range 7–55 mm) and the mean adenoma estimated volume was 6.65 cm^3^ (range 0.101–40.068). Suprasellar extension and stalk deviation was evident in 73 and 89%, respectively. CS invasion was present in 60%. On T1-weighed images, 72.5% were isointense, 20% were hypointense, and 7.5% were hyperintense; on T2-weighed images, 71.6% were hyperintense, 22.5% were isointense, and only 5.8% were hypointense. Post-gadolinium enhancement was present in 85%, while a heterogeneous parenchymal appearance was seen in 62% (Table 1A).

### Hormonal deficits

Our primary outcome, secondary adrenal insufficiency, central hypothyroidism, or both (SAI/CHT), was documented in 54 patients (45%), distributed as follows: 29 patients (24%) had SAI, 48 patients (40%) had CHT, and 24 patients (20%) had both SAI and CHT. Thus, 14% of patients with SAI did not have CHT while 50% of patients with CHT did not have SAI. Isolated SAI/CHT (i.e. with intact remaining pituitary hormones) was seen in only six patients (5% of all patients, 11% of those with SAI/CHT). Forty-eight patients (89%) with SAI/CHT had at least one other hormonal deficit, 32 patients (59%) had at least two other hormonal deficiencies. Sixty-five percent of all patients had at least one pituitary hormone deficiency detected at diagnosis (any pituitary hormonal deficit); 45% had at least 2, and 32% had at least three pituitary hormone deficits (Table 2).

**Table 2:** Distribution of hypopituitarism in 121 patients with NFPA.

Hormonal deficit	*n* (%)
	
Any hormonal deficit	79 (65%)
At least two hormone deficits	55 (45%)
At least three hormone deficits	39 (32%)
LH/FSH	69 (57%)
ACTH and/or TSH	54 (45%)
ACTH	29 (24%)
TSH	48 (40%)
ACTH and TSH	24 (20%)

ACTH, adrenocorticotropic hormone; FSH, follicle stimulating hormone; LH, lutenizing hormone; TSH, thyroid stimulating hormone.

### Predictors of hypopituitarism

In our multivariate analysis, older age was significantly associated with SAI/CHP (*P =* 0.0206) (Table 1B). The mean age for patients with SAI/CHT was 7 years older than for patients without these hormonal deficits (60.4 vs 53.4 years of age). Further stratification by age groups showed that 70.4% of patients with SAI/CHT were older than 55 years, while 70% of patients without SAI/CHT were younger than 65 years. Of note, the average adenoma size did not appear to increase with age (Supplementary Table 1, see section on supplementary materials given at the end of this article). In terms of sex distributions, male patients had a higher prevalence of SAI/CHT (52.1%) in comparison with female patients (33.3%, *P = *0.043). Seventy percent of patients with SAI/CHT were males (Table 1B).

Of the MRI characteristics that do not denote direct measurement of tumor size, stalk deviation, optic chiasma compression, and adenoma post-gadolinium enhancement were significantly associated with SAI/CHT. Neither CS invasion, tumor heterogeneity, nor MR-signal intensity were predictive of SAI/CHT (Table 1B). Of patients with SAI/CHT, 100% had stalk deviation (*P* < 0.0001); 86.7 vs 62.7% (*P = *0.012) were in contact or displacing the optic chiasma (OC), and 94.4 vs 77.6% (*P = *0.029) had gadolinium enhancement (Table 1B).

Adenoma size measured by vertical height, largest diameter or estimated volume was also significantly associated with SAI/CHT. This association remained significant for all three measurements in a multivariate analysis, with similar OR for vertical height (OR 1.1, 95% CI 1.03–1.15, *P* < 0.0001), largest diameter (OR 1.09, 95% CI 1.03–1.16, *P = *0.005), and adenoma volume (OR 1.10, 95% CI 1.00–1.21, *P = *0.035) (Table 3). There was 100% sensitivity for detection of SAI/CHT at vertical height 12 mm, largest diameter 17 mm, and estimated volume 0.95 cm^3^ (Table 4).

**Table 3:** Logistic regression to predict the presence of SAI and/or CHT by vertical height, adenoma largest diameter, and adenoma volume.

Variable	Vertical height		Largest diameter		Adenoma volume	
	OR (95% CI)	*P*-value	OR (95% CI)	*P*-value	OR (95% CI)	*P*-value
Size measure	1.10 (1.03–1.15)	<0.0001	1.09 (1.02–1.16)	0.008	1.10 (1.00–1.21)	0.035
Age	1.03 (1.00–1.06)	0.030	1.03 (1.00–1.06)	0.034	1.04 (1.00–1.06)	0.020
Sex	1.96 (0.84–4.55)	0.125	2.01 (0.85–4.73)	0.111	1.96 (0.84–4.55)	0.119
CS invasion	0.83 (0.34–1.94)	0.598	0.75 (0.31–1.80)	0.516	0.81 (0.35–1.91)	0.636
Homogenity	0.74 (0.31–1.73)	0.512	0.69 (0.29–1.64)	0.401	0.75 (0.39–1.76)	0.504
Enhancement	1.00 (0.97–1.04)	0.734	1.00 (0.97–1.04)	0.779	1.00 (0.97–1.04)	0.825
Cystic	0.69 (0.23–2.09)	0.504	0.67 (0.22–2.05)	0.489	0.68 (0.22–2.10)	0.505
OC compression	1.07 (0.67–1.65)	0.788	1.24 (0.67–2.25)	0.487	1.42 (0.80–2.54)	0.227

CS, cavernous sinus; OR, odds ratio; OC, optic chiasma.

**Table 4:** Sensitivity, specificity, and likelihood ratio (LR+) for adrenal insufficiency and/or central hypothyroidism with different cut-off points where the three measurements of tumor size were evaluated.

Adenoma craniocaudal diameter					Adenoma largest diameter					Adenoma estimated volume					
Cutpoint (mm)	Sensitivity (%)	Specificity (%)	Correctly classified (%)	LR+	Cutpoint (mm)	Sensitivity (%)	Specificity (%)	Correctly classified (%)	LR+	Cutpoint (cm^3^)	Equivalent mean diameter	Sensitivity (%)	Specificity (%)	Correctly classified (%)	LR+
**≥12**	100.00	20.90	56.20	1.2642	**≥17**	100.00	26.87	59.50	1.3673	**≥0.95**	12.3 mm	100.00	20.90	56.20	1.2642
**≥14**	98.15	31.34	61.16	1.4295	**≥19**	94.44	35.82	61.98	1.4716	**≥1.45**	14.2 mm	98.15	31.34	61.16	1.4295
**≥16**	92.59	38.81	62.81	1.5131	**≥20**	92.59	40.30	63.64	1.5509	**≥1.94**	15.7 mm	92.59	35.82	61.16	1.4427
**≥18**	92.59	44.78	66.12	1.6767	**≥21**	87.04	43.28	62.81	1.5346	**≥2.77**	17.7 mm	87.04	46.27	64.46	1.6199
**≥20**	83.33	49.25	64.46	1.6422	**≥22**	79.63	47.76	61.98	1.5243	**≥3.26**	18.6 mm	81.48	53.73	66.12	1.7611
**≥22**	66.67	55.22	60.33	1.4889	**≥23**	77.78	50.75	62.81	1.5791	**≥3.62**	19.3 mm	75.93	55.22	64.46	1.6957
**≥24**	59.26	65.67	62.81	1.7262	**≥25**	62.96	58.21	60.33	1.5066	**≥4.19**	20 mm	66.67	58.21	61.98	1.5952
**≥26**	55.56	70.15	63.64	1.8611	**≥27**	55.56	70.15	63.64	1.8611	**≥5.01**	22 mm	55.56	64.18	60.33	1.5509
**≥28**	50.00	77.61	65.29	2.2333	**≥30**	48.15	79.10	65.29	2.3042	**≥5.98**	22.8 mm	51.85	70.15	61.98	1.7370
**≥30**	42.59	85.07	66.12	2.8537	**≥34**	31.48	88.06	62.81	2.6366	**≥7.19**	24.3 mm	46.30	76.12	62.81	1.9387
**≥34**	29.63	91.04	63.64	3.3086	**≥36**	20.37	88.06	57.85	1.7060	**≥9.86**	27 mm	37.04	85.07	63.64	2.4815
**≥38**	14.81	95.52	59.50	3.3086	**≥39**	11.11	95.52	57.85	2.4815	**≥13.27**	29.8 mm	16.67	91.04	57.85	1.8611
**≥42**	5.56	98.51	57.02	3.7222	**≥42**	5.56	98.51	57.02	3.7222	**≥19.54**	33.9 mm	7.41	98.51	57.85	4.9630
**≥45**	5.56	100.00	57.85		**≥45**	5.56	100.00	57.85		** ≥19.99**	34 mm	7.41	100.00	58.68	

LR, likelihood ratio.

The calculated size cut-off where risk of having SAI/CHT significantly increased was calculated using ROC. It was 18 mm for vertical height (sensitivity 92.6%, specificity 44.8%, PPV 57.5%, and NPV 88.2%); 23 mm for adenoma largest diameter (sensitivity 73.9%, specificity 56.0%, PPV 77.8% and NPV 50.7%); and 3.23 cm^3^ for estimated tumor volume (sensitivity 78.3%, specificity 58.7%, PPV 81.5%, and NPV 53.7%) (Tables 4 and 5).

**Table 5:** Statistical analysis of cut off sizes of vertical height, largest diameter, and adenoma volume that predicts the presence of central hypopituitarism.

Cut-off size	Vertical height	Largest diameter	Estimated volume
	>18.00 mm	>23.00 mm	>3.23 cm^3^
Sensitivity	92.59%	73.9%	78.3%
Specificity	44.78%	56%	58.7%
ROC area	0.734	0.710	0.703
Positive LR	1.68	1.58	1.76
Negative LR	0.165	0.44	0.34
Odds ratio	10.1	3.61	5.11
PPV	57.5	77.8	81.5
NPV	88.2	50.7	53.7

LR, likelihood ratio; NPV, negative predictive value; PPV, positive predictive value; ROC, receiver operating characteristic.

## Discussion

In this series of 121 patients with NFPA, hypopituitarism was positively associated mainly with larger adenoma size (Table 1B). Therefore, our finding supports the notion that a decision on the extent of laboratory testing for detecting hypopituitarism in patients with non-functioning pituitary adenomas is best based on tumor size. However, of great interest, we also found other factors that were also positively related to the more severe pituitary hormonal deficiencies, namely ACTH and TSH, which may contribute significantly to the decision-making process. We found that older age, male sex, and MRI findings of optic chiasma compression, pituitary stalk deviation, and adenoma enhancement post-gadolinium were also predictive of these hormonal deficiencies.

In our study, we assessed adenoma size using three different measurements, namely: (i) vertical height (craniocaudal diameter); (ii) longest of craniocaudal, anteroposterior, and transversal diameters; and (iii) estimated adenoma volume; all of these measurements were found to be positively associated with the presence of SAI/CHT. After controlling for the potential confounders included in our data, the association was still significant. However, the vertical height was the most reliable predictor of both SAI and CHT, and the only one significantly associated with any one or more pituitary hormonal deficiency in a multivariate analysis (Table 6). Of note, previous studies have mostly used either maximum (16) or mean adenoma diameter (19) as predictors of hypopituitarism.

**Table 6:** Logistic regression to predict the presence of any one hormonal deficiency by vertical height, largest diameter, and adenoma volume.

Variable	Vertical height			Largest diameter			Adenoma volume		
	Univariate, OR (95% CI)	Adjusted, OR (95%)	*P*-value	Univariate, OR (95% CI)	Adjusted, OR (95%)	*P*-value	Univariate, OR (95% CI)	Adjusted, OR (95%)	*P*-value
Size measure	1.08 (1.03–1.12)	1.06 (1.01–1.12)	0.001	1.07 (1.02–1.12)	1.05 (0.99–1.12)	0.105	1.12 (1.02–1.22)	1.09 (0.98–1.20)	0.118
Age		1.03 (1.00–1.06)			1.03 (1.00–1.06)			1.03 (1.00–1.06)	
Sex		2.10 (0.91–4.85)			2.12 (0.93–4.86)			2.14 (0.94–4.90)	
Invasiveness		0.93 (0.38–2.24)			0.94 (0.39–2.28)			0.99 (0.42–2.34)	
Homogenity		0.97 (0.41–2.29)			0.94 (0.40–2.22)			0.98 (0.42–2.30)	
Enhancement		0.99 (0.97–1.02)			1.00 (0.97–1.03)			0.99 (0.96–1.02)	
Cystic		1.06 (0.67–1.70)			1.07 (0.67–1.70)			1.08 (0.58–1.99)	
OC compression		1.04 (0.83–1.28)			1.14 (0.63–2.06)			1.18 (0.67–2.08)	

OR, odds ratio; OC, optic chiasma.

In our study, we focused on deficiency of ACTH and/or TSH. We looked at their deficits when diagnosed before any medical or surgical intervention because of the relevant practical implications in the short term, particularly when surgery is planned. Of great interest for practical reasons, we found that these hormonal deficiencies, not only did not occur in patients with microadenomas, but actually did not occur in any of our patients whose adenoma maximum diameter was under 17 mm (Table 4), which is well over the 10 mm cut-off point for separating micro- from macroadenomas.

Our findings also show that when the vertical (craniocaudal) diameter is used for adenoma size estimation, the point at which SAI/CHT becomes a concern (92.6% sensitivity) is also around the 18 mm cut-off. SAI/CHT was not detected in our patients with tumors with craniocaudal diameter of 12 mm or less, largest diameter of 17 mm or less, or estimated volumes below 1 cm^3^ (Table 4). SAI/CHT rarely happened when vertical diameter was less than 18 mm, when the adenoma largest diameter was under 20 mm, or when the estimated linear volume was under 3.0 cm^3^. An OR of around 1.1 for the probability of SAI/CHT with any measure of tumor size (Table 3) means that for every unit increase in any tumor size indicator (in mm for vertical height and cm^3^ for volume) there is a 10% increase in the probability of SAI/CHT after controlling for the other variables investigated. For instance, for every 1 cm increase in largest diameter, the likelihood of adrenal insufficiency, central hypothyroidism, or both doubled.

In several series, hypopituitarism, in general, is reported in 41–89% of NFPA (15, 20). The most frequent hormonal deficit is usually GH (70–80%), followed by hypogonadotropic hypogonadism (40–75%). Central hypothyroidism and hypocortisolism occur less frequently, in approximately 20–40% of cases (21, 22). Hypopituitarism is rare in microadenomas in most series, with few exceptions including reports by Wichers-Rother and Yuen *et al.* (23, 24) where an impressive 50–80% of microadenomas had GH deficit, 24–38% had gonadotropin deficiency, 12–40% had TSH deficiency, and 4–28% had ACTH deficiency. In these studies, a large percentage of patients were referred to tertiary care centers because of abnormal pituitary function or symptoms of hypopituitarism, which may represent a referral bias, of a specific subgroup of microadenoma carriers. SAI/CHT was seen in a relatively large number (45%) of cases in our series, which is not surprising as 93% of patients in our series had macroadenomas and mean adenoma largest diameter was 25.7 mm.

Our results are in line with several previous studies. Fernández-Balsells *et al*. reported an increased incidence of new endocrine dysfunction in macroadenoma (11.9 per 100 person-year; 95% CI 0.0–30.8) compared with microadenoma (4.0 per 100 person-years; 95% CI 0.0–6.4) (25). Jahangiri *et al*. (19) studied 305 patients with NFPA and found 50% preoperative hypopituitarism. Patients with hypopituitarism had significantly larger adenomas (24 vs 21 mm; *P* = 0.02). A higher prevalence of hypopituitarism in larger NFPA was reported as well by Mukai *et al*. (16) in 63 NFPA. In this series, the frequency of hypopituitarism (deficit of either ACTH, TSH, GH, or LH/FSH) was 31%, in tumors 1–19 mm, 61% in tumors 20–29 mm, and 82% in tumors >30 mm in largest diameter (*P* < 0.01); multi-hormonal deficiency was also associated with larger tumors (*P* < 0.005). Similar to our series, they found no cases of SAI/CHT in tumors smaller than 19 mm in diameter.

Our study showed that at adenoma volume cut-off of 3.23 cm^3^ the PPV for SAI/CHT was 81.5% (sensitivity 78.3%, specificity 58.7%). Jahangiri *et al*. found an association between any pituitary hormonal deficit and an adenoma volume of 7.3 cm^3^ (*P* = 0.009), a much higher cut-off (19). However, while these authors measured tumor volumes using the AWServer software from GE Healthcare, we used the simplified ellipsoid formula, which lacks the precision of semiautomatic methods that take into account the irregularities and intricacies of these tumors (26). The advantage of our approach is that it is readily accessible to any clinician with access to MR images. It is noteworthy that, although we did not measure it in the present study, the volume of the normal pituitary gland surrounding the adenoma has been assessed by others, and no correlation was found with preoperative hypopituitarism in NFPA (19).

In our study, an adenoma vertical height over 18 mm predicted SAI/CHT with a sensitivity of 92.6% (specificity of 44.8%, NPV 88%). Similarly, adenoma largest diameter over 23 mm predicted SAI/CHT with a sensitivity of 73.9% (specificity 56%, PPV 77.8%). Our results are comparable to those of Mukai *et al*., who found that a maximum adenoma diameter ≥ 24.2 mm predicted CHT with a sensitivity of 83.3% and a specificity of 47.7% (16).

In regards to tumor characteristics associated with tumor tissue qualities, which included signal intensity in T1 and T2, signal heterogeneity and post-contrast injection enhancement, only post-contrast enhancement was significantly associated with secondary adrenal insufficiency, central hypothyroidism or both. Overall, 72% of the adenomas that we studied had isointense signal intensity in T1-weighed images; 71% had hyperintense signal intensity on the T2-weighed image. Of note, MRI signal intensity reflects the density of the secretory granules in functioning lesions and is of prognostic value especially in the case of somatotroph adenomas, where T2 hypointensity (associated with a densely granulated histology pattern) is associated with better response to somatostatin analogs (14, 27). A recent study evaluated this MRI feature in other functioning adenomas and found that T2-hypointensity in female prolactinomas was related to younger age at diagnosis, higher baseline prolactin (PRL) levels, and dopamine agonist resistance. In the case of corticotroph adenomas, T2- hyperintensity is seen in larger lesions with the sparsely granulated cytoplasmic pattern (14).

Post-contrast (gadolinium) enhancement, a reflection of the adenoma capillary vascular density, is typically lower in pituitary adenomas compared with normal pituitary. It tends to be lower in NFPA compared with secreting adenomas (13). However, this feature was not studied previously as a predictor of pituitary dysfunction. In our series, a majority (87%) of NFPA showed enhancement after gadolinium administration. After correcting for tumor size and other parameters, gadolinium enhancement remained positively associated with SAI/CHT, occurring in 94.4% of patients with SAI/CHT vs 77.6% of those without SAI/CHT (*P* = 0.029) (Table 2). It remains to be determined whether using an automatic method of quantification of contrast enhancement would result in a stronger association.

In regards to the MRI characteristics related to tumor invasiveness, our study showed a significant association of SAI/CHT with both stalk deviation and OC compression, but not with CS invasion. In our series, no adenoma without stalk deviation had severe hormonal deficits. These associations were not seen by Mukai *et al*., who found that stalk deviation was associated with GH deficiency but not ACTH deficiency. This difference may be because they excluded in their analysis tumors with suprasellar extension when the stalk was not visible (16). For us, such tumors were assumed to have stalk deviation. For Mukai *et al*., the presence of visual field defects correlated only with GH deficiency (16).

CS invasion was not associated with SAI/CHT in our study. In previous reports, CS invasion was found to be related to GH deficiency and male hypogonadism but not with SAI/CHT (16). In a retrospective study of 390 NFPA by Nishioka *et al*., CS invasion was significantly more common in certain NFPA subtypes, namely silent ACTH, GH, TSH, and PRL adenomas compared with null cells or gonadotroph adenomas (28).

Finally, our association analysis showed that older age and male sex were positively associated with SAI/CHT. However, only older age remained statistically significant in our multivariable analysis. Of note, while male patients tend to have larger adenomas, there was no difference in mean adenoma size in the different age groups (Supplementary Table 1). Jahangiri *et al*. findings were in line with ours in regards to male sex and older age correlation with hypopituitarism (17).

Given that our data were obtained retrospectively from a single tertiary care practice, our conclusions require to be revisited prospectively in a larger series. In the meantime, it seems reasonable to alert neuroendocrinologists and neurosurgeons to be vigilant and assure that patients with NFPA who meet the size criteria reported here (vertical height over 18 mm, adenoma largest diameter over 23 mm, and adenoma volume over 3.2 cm^3^) must undergo static and dynamic testing to rule out both adrenal insufficiency and central hypothyroidism, as soon as possible and certainly before any surgical intervention. In addition, in emergency situations where neurosugeons do not have the time to perform dynamic testing, such adenoma size cut-offs could alert them to proceed with empirical perioperative glucocorticoid coverage due to the higher risk of SAI in the setting of such findings.

Equally relevant in clinical practice is the finding in our series that the likelihood of hypopituitarism involving the adrenal and thyroid axis is minimum for small macroadenomas, that is, adenomas with a craniocaudal diameter below 12 mm, maximum diameter less than 17 mm, and estimated volume ((APxCCxT)/2) less than 0.9 cm^3^. Such patients can probably undergo surgery after just static hormonal testing, in addition to the appropriate tests to rule out hormonal oversecretion. In fact, AM cortisol is predictive of post-ACTH-stimulated cortisol level especially when it is above 243–266 nmol/L which is correlated with post-ACTH-stimulated cortisol of above 500-550 nmol/L, respectively (29). In practice, this can be applied as for patients with adenomas smaller than the 17 mm in maximum diameter, only patients with AM cortisol below such cut-off and/or symptoms of adrenal insufficiency would undergo stimulation tests. This has to be tempered as well with the fact that ACTH stimulation tests, both low and high dose, are fraught with sensitivities and specificities around 80–90% at best (30).

Interestingly, the cut-off for estimated volume under which is rare to find SAI/CHT in NFPA appears relatively low (0.9 cm^3^) in relation with largest diameter and height cut-offs. This apparent incongruency suggests that macroadenomas that tend to be spheroidal (all diameters similar to largest diameter) have a higher risk of SAI/CHT, compared with those with a flatter ellipsoidal shape.

Our study involved a good number of NFPA patients, showed relevant important results which could help in the care of NFPA patients. In addition, we looked at other clinical and MRI characteristics which could predict the presence of hypopituitarism. Furthermore, we provided cut-off adenoma sizes which could help clinicians to predict the need for dynamic testing. However, given that our series is retrospective and from single tertiary center, further prospective multicenter studies with greater number of patients are required to revisit our findings.

## Conclusion

According to our data, the main predictors of secondary adrenal insufficiency and central hypothyroidism in NFPA are larger tumor size, suprasellar extension causing stalk deviation, and optic chiasma compression. For the estimation of adenoma size, either vertical adenoma height, adenoma's largest diameter, or adenoma volume can be used. A vertical adenoma height over 18 mm, adenoma's largest diameter over 23 mm, and adenoma estimated volume over 3.2 cm^3^ should compel clinicians to proceed with dynamic testing to rule out secondary adrenal insufficiency. Once adrenal insufficiency is ruled out or confirmed and treated, then assessment for secondary hypothyroidism should be reassessed. Clinical evaluation, along with measurement of free T4 and TSH, will dictate the need for levothyroxine replacement. Additionally, male patients, older patients, and patients whose tumors enhance with gadolinium are at higher risk of SAI/CHT.

## Supplementary Material

Supplementary Table 1: Mean different adenoma sizes by different age groups and sex. Abbreviations: cm<sup>3</sup>: cubic centimeter, mm: millimeter.

## Declaration of interest

The authors declare that there is no conflict of interest that could be perceived as prejudicing the impartiality of the research reported.

## Funding

This work did not receive any specific grant from any funding agency in the public, commercial, or not-for-profit sector.

## Author contribution statement

Reem Al Argan and Abdulaziz Ramadhan collected the data. Ramanakumar V Agnihotram contributed in the statistical analysis and reviewed the paper from statistics part. Jeffrey Chankowsky contributed in reviewing the MRIs of the data population. Juan Rivera supervised the whole process. Reem Al Argan and Juan Rivera wrote the manuscript. All authors have read the manuscript and approved it.
