# Supplementary material for: Baseline MRI findings as predictors of hypopituitarism in patients with non-functioning pituitary adenomas
Source: Endocr Connect. 2021 Oct 11;10(11):1445–54. doi: 10.1530/EC-21-0386 (PMC8630757; doi:10.1530/EC-21-0386)
Supplement: Supplementary Table 1: Mean different adenoma sizes by different age groups and sex. Abbreviations: cm3: cubic centimeter, mm: millimeter. [file supplementary_table_1.pdf]

**Variable**	**Size Indicator**		
**Age Group**	**Mean Adenoma Volume (cm^3)**	**Mean Vertical Height (mm)**	**Mean Largest Diameter (mm)**
<55	6.53	22.2	24.1
55-64	5.79	24.0	25.8
65-74	6.68	25.4	27.9
75+	6.85	23.8	25.5
**Sex**			
Females	5.7	21.9	24.1
Males	6.9	24.4	26.1

Supplementary Table 1: Mean different adenoma sizes by different age groups and sex.
Abbreviations: cm^3: cubic centimeter, mm: millimeter.
